# Awareness of the Saudi population regarding the effects of smoking on fracture healing

**DOI:** 10.18332/tid/207130

**Published:** 2025-08-21

**Authors:** Hamza M. Alrabai, Abdulmalik Alduraibi, Abdulaziz Alrabiah, Saad Al Ghadir, Khalid Alghamdi, Khalid Alhamdi, Abdulaziz Bahammam, Waleed Albishi

**Affiliations:** 1 Department of Orthopedic Surgery, College of Medicine, King Saud University, Riyadh, Saudi Arabia; 2 College of Medicine, King Saud University, Riyadh, Saudi Arabia

**Keywords:** smoking, fracture healing, knowledge, cross-sectional studies, healthcare costs

## Abstract

**INTRODUCTION:**

Smoking tobacco negatively affects fracture healing, increasing the risk of delayed union, malunion, and nonunion, as well as healthcare expenses. Although smoking is common in Saudi Arabia, the literature on public awareness of the negative impact of smoking on fractures is limited. Our study aimed to address this gap and assess the population’s knowledge on the effects of smoking on fracture healing.

**METHODS:**

An analytical cross-sectional study, involving 1033 Saudi adults, was conducted between June and September 2024 in Saudi Arabia. A validated and reliable self-created Smoking and Fracture Knowledge Assessment tool (SFKAT) was used in an online questionnaire to collect the participants' responses. Participants were categorized into good- or poor-knowledge groups based on the median SFKAT score. Binary logistic regression was used to adjust for confounding variables.

**RESULTS:**

The participants included 54.4% men. The median age of the participants was 39 years and 24.1% of them were smokers. Most smokers were men (87%). The median SFKAT score was 16 (interquartile range, IQR=12–19) and 53% of the respondents demonstrated good knowledge on the effects of smoking on fracture healing. The likelihood of good-knowledge scoring was considerably lower for men than women (adjusted odds ratio, AOR=0.48: 95% CI: 0.36–0.65; p<0.001). Healthcare workers were more likely to have a good-knowledge scoring (AOR=2.78; 95% CI: 1.90–4.08; p<0.001). Smokers had lower odds of having a good-knowledge scoring (AOR=0.54; 95% CI: 0.38–0.75; p<0.001).

**CONCLUSIONS:**

The awareness of the detrimental effects of smoking on fracture healing is suboptimal, particularly among men and smokers. Sex, healthcare work, and smoking were significantly associated with knowledge levels after adjusting for confounders. These findings may encourage educational strategies and direct counseling for populations with an observed knowledge gap to help lower the burden and treatment costs.

## INTRODUCTION

Bone healing occurs following a bone fracture. As described by ElHawary et al.^1^, this process is categorized as either primary or secondary bone healing. Primary bone healing occurs when the distal and proximal bone fragments are perfectly reduced and aligned under compression^1^.

Secondary bone healing is a more common process in which the bone heals indirectly in a four-stage process: formation of hematoma, fibrocartilaginous callus, and bony callus, followed by bone remodeling^1^.

If the process of bone healing takes >12 weeks, it is defined as a delayed union fracture^2^. Furthermore, in the absence of radiological signs of bone healing for 10 weeks post-injury, the fracture is classified as a nonunion fracture^3^. However, if bone healing occurs and the bone heals in an abnormal position, the term malunion is used to describe the fracture^3^.

These impairments in bone healing increase the duration of hospital stay and treatment costs. Fracture-healing complications were the cause of re-admission in 8.1% of patients treated for bone fractures in a study of 3886 patients and these re-admissions accounted to $4.9 million Australian Dollars^4^. Moreover, the cost of treating a long bone nonunion averaged $11800, US$11333, and £29204 in Canada, the USA, and the UK, respectively^5^.

Cigarette smoking negatively affects bone healing. Through vasoconstriction, nicotine decreases blood perfusion to target tissues, such as muscles, tendons, ligaments, and bones, thereby impairing bone healing^6^. A 2022 study found that cigarette smoking increased the risk of fracture nonunion by 91% in spine surgeries and 104% in autograft recipients^7^. According to a study conducted in Riyadh by Aldhafian et al.^4^, smoking was the most common patient-dependent risk factor associated with fracture-healing failure. This effect appears to apply to e-cigarettes as well; one study showed that e-cigarette users have a 46% higher prevalence of self-reported fragility fractures^8^.

According to the Global Adult Tobacco Survey, 17.9% of the Saudi population are smokers, with a prevalence of 27.5% among men and 3.5% among women^9^. A 2019 study found that the prevalence of tobacco smoking among Saudi university students was 17%^10^. The World Health Organization states that 22.3% of the global population uses tobacco^11^.

Smoking exerts harmful effects on bone health. It increases the risk of osteoporosis and delays bone healing in fracture settings^12,13^. Various studies have highlighted the need to raise public awareness of its risks^14,15^.

Studies on the Saudi population’s perceptions of the effects of smoking on fracture healing are lacking, which are important considering the burden of delayed fracture healing due to the high prevalence of smoking. To address this issue, our study aimed to provide local original data to build a more comprehensive understanding of the population’s knowledge on the effects of smoking on fracture healing. Access to such data will facilitate the filling of patient knowledge gaps by healthcare providers.

## METHODS

### Data, study population, and variables

This cross-sectional study was conducted between June and September 2024 in Saudi Arabia. Saudi individuals aged ≥18 years were included in this study. A total of 1033 participants filled in the questionnaires electronically via social media platforms.

The questionnaire consisted of 32 questions divided into three sections. The first part comprised sociodemographic data. The second part included our validated and reliable Smoking and Fractures Knowledge Assessment Tool (SFKAT) (Supplementary file Appendix 1). This tool consists of ten questions. Each question can be answered by ‘true’, ‘not sure’, or ‘false’. A correct answer is calculated as two points, ‘not sure’ is calculated as one point, and an incorrect answer is calculated as zero points. The maximum total score is 20 points. The median score was used to classify the participants into good- and poor-knowledge groups, with participants receiving scores at or above the median classified into the good-knowledge group. The third section of the questionnaire aimed to obtain information on the primary sources of knowledge that the participants used to learn about smoking and fractures. This approach was implemented in a study by Alshammari et al.^16^. Forward and back-translations were used to translate the survey. We then conducted a pilot study with 45 participants and added open-ended questions at the end of each section to assess the simplicity and clarity of each part and correct any misunderstandings. The questionnaire was then distributed through social media platforms to the participants using snowball sampling.

*A priori* sample-size calculations and justifications were based on two approaches. G*Power was used for the binary logistic regression (Wald test). We assumed a medium effect size (OR=1.5), α=0.05, power=0.80, and R^2^=0.1. This resulted in a minimum sample size of 231 participants, which was required to detect a significant association for a single predictor. The 10 events per variable rule was then applied to confirm the stability of the multivariable binary logistic regression model. With 14 predictors and 549 participants classified as having ‘good knowledge’, the final sample of 1033 participants exceeded both criteria, supporting the adequacy of the sample size for a robust analysis.

### Validity and reliability of the SFKAT

A specialist in orthopedic surgery confirmed that the questions were clear and accurate and that they aligned with our primary objective. The tool showed excellent internal consistency, with a split-half reliability of 0.87 and a Spearman-Brown coefficient of 0.93. Confirmatory factor analysis showed moderate construct validity with acceptable model fit indices (comparative fit index=0.92, Tucker-Lewis index=0.9, and root mean square error of approximation=0.099). Five of ten items of the SFKAT had factor loadings exceeding 0.4, indicating partial structural validity. These results suggest that the SFKAT is a moderately valid and highly reliable instrument to assess knowledge in this population.

### Ethics

The study was approved on 10 June 2024, by the Institutional Review Board of the King Saud University with reference number E-24-8893. Consent was obtained from all participants before they completed the questionnaire. The respondents’ anonymity was maintained. The data were used exclusively for research purposes.

### Statistical analysis

IBM SPSS Statistics software for Windows (version 21.0; IBM Corp., Armonk, N.Y., USA) was used to analyze the data. Categorical variables are described using descriptive statistics (frequency and percentage). Continuous variables are described using median and interquartile range (IQR). The relationship between categorical study variables and outcome variables was analyzed using Pearson’s chi-squared test. ORs were used as indicators of the association between two categorical variables. For multivariate analysis, a binary logistic regression model was implemented to identify the independent predictors of knowledge levels. The process of variable selection in the model was based on a combination of statistical significance and theoretical relevance. Variables with a p<0.2 in the univariate analysis were included. Age and sex were included *a priori* because of their known influence on health-related knowledge. Nineteen variables were included in the final model. Adjusted ORs (AORs) with 95% confidence intervals (CIs) are reported. Model fit was tested using the Hosmer-Lemeshow goodness-of-fit test. Explanatory power was evaluated using Cox and Snell R^2^ and Nagelkerke R^2^ statistics. The analyzed data are displayed using tabular and graphical presentation methods. The study reported data precision through 95% CIs and statistical significance levels at p<0.05. To assess the robustness of the median-based knowledge classification, a sensitivity analysis was conducted using alternative cut-off values: the upper 33rd percentile (score ≥18) and the upper 25th percentile (score ≥19). Binary logistic regression was repeated using these alternative definitions of ‘good knowledge’, and model consistency was compared.

## RESULTS

### Sociodemographic characteristics and descriptive statistics

The total sample consisted of 1033 participants aged 18–85 years. Age distribution was positively skewed, with a median age of 39 years (IQR=25–50). Among the participants, 54.4% were men. In addition, 29% of participants reported a history of fractures. Among the sample, 249 participants (24.1%) reported smoking behaviors, including cigarettes, vaping, or shisha (Supplementary file Appendix 2). Approximately 87% of smokers were men. The prevalence of smoking was 38.4% in men and 7% in women. In addition, 61% of the smokers were aged 26–50 years.

### Descriptive statistics of the total scores

**Figure 1:**
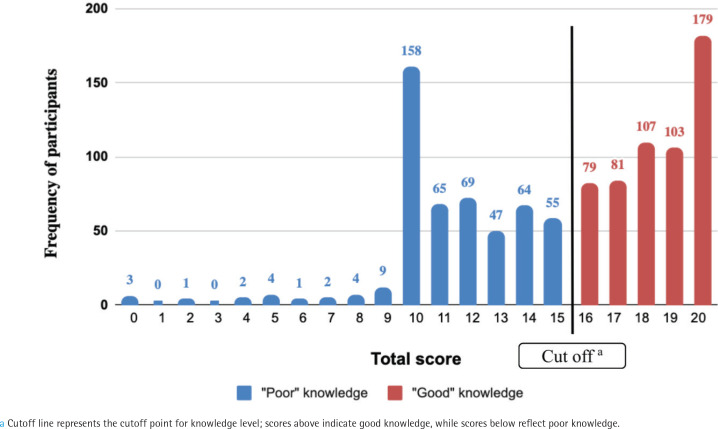
Distribution of total SFKAT scores among participants, a cross-sectional study conducted in Saudi Arabia, June–September 2024 (N=1033)

The total scores obtained from the SFKAT followed a non-parametric negatively skewed distribution (Figure 1), with a median of 16 (IQR=12–19). Almost 53% of participants had good levels of knowledge. Female participants had a median total score of 17 (IQR=14–19). Most of the women had a good level of knowledge (64.5%). For men, the median total score was 14 (IQR=10–18), with only 43.6% scoring a good level of knowledge. The median total score of smokers was 13 (IQR=10–17), whereas that of non-smokers was 17 (IQR=12–19). Only 35% of smokers scored a good-knowledge level compared with 59% of non-smokers.

### Main sources of knowledge

**Figure 2:**
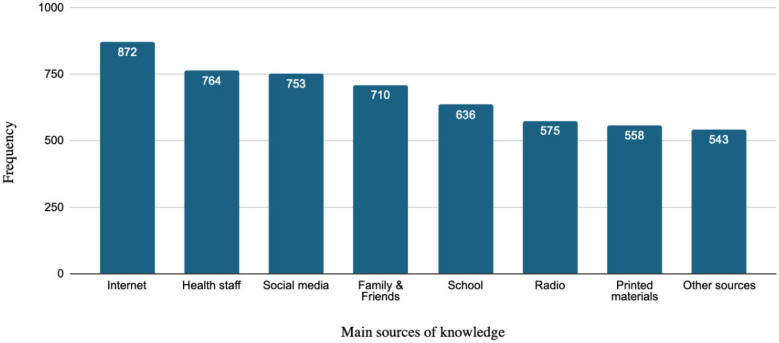
Frequency of choosing the main sources of knowledge, a cross-sectional study conducted in Saudi Arabia, June–September 2024 (N=1033)

Regarding knowledge acquisition, participants relied predominantly on the internet (84%) and healthcare personnel (74%) as their main sources of information. The least common sources included radio, printed materials, and ‘other sources’ (Figure 2).

### Association between sociodemographic characteristics and level of knowledge

The odds of having a good level of knowledge were lower in men than in women (OR=0.43; 95% CI: 0.33–0.55; p<0.001). The odds of having a good level of knowledge were 2.29 times greater among participants working in healthcare fields than those in non-health professions (95% CI: 1.66–3.17; p<0.001). The participants with a history of fracture had 29% lower odds of acquiring a good level of knowledge compared to those who did not have a fracture history (95% CI: 0.55–0.93; p=0.014). Regarding smoking status, the analysis reported smoking cigarettes, shisha, or vaping was associated with a lower likelihood of having a good level of knowledge compared to non-smokers (OR=0.38; 95% CI: 0.29–0.52; p<0.001) (Table 1).

**Table 1:** Association between the dichotomous sociodemographic characteristics and the level of knowledge of the Saudi population, a cross-sectional study conducted in Saudi Arabia, June–September 2024 (N=1033)

*Characteristics*	*Level of knowledge*			*p*	*OR*	*95% CI*
	*Good Score ≥16*	*Poor Score ≤15*	*Total*			
	*n (%)*	*n (%)*	*n (%)*			
**Sex**						
Male	245 (43.6)	317 (56.4)	562 (100)	<0.001	0.43	0.33–0.55
Female	304 (64.5)	167 (35.5)	471 (100)			
**Working in the health field**						
Yes	144 (68.9)	65 (31.1)	209 (100)	<0.001	2.29	1.66–3.17
No	405 (49.2)	419 (50.8)	824 (100)			
**Having a fracture**						
Yes	142 (47.2)	159 (52.8)	301 (100)	0.014	0.71	0.55–0.93
No	407 (55.6)	325 (44.4)	732 (100)			
**Smoking cigarettes, shisha, or vape**						
Yes	88 (35.3)	161 (64.7)	249 (100)	<0.001	0.38	0.29–0.52
No	461 (58.8)	323 (41.2)	784 (100)			

Age groups were significantly related to the level of knowledge, as the respondents who had a good level of knowledge in those aged ≥51 years, were significantly lower than the expected count of respondents, while all other age groups had higher counts than expected (p=0.048). No significant association was noted between the level of knowledge and any other polytomous demographic variables.

### Association between main sources and level of knowledge

Using the internet as a primary source of knowledge was not significantly related to the level of knowledge. However, all remaining sources had a statistically significant association with the level of knowledge, with varying ORs (Table 2). Participants who chose school as their source of information were 1.03 times more likely to have a good level of knowledge. Additionally, participants who sought healthcare personnel as knowledge source were 1.83 times more likely to have a good level of knowledge. Participants who utilized ‘other sources’ to gain knowledge had the highest likelihood of having a good level of knowledge (OR=2.04).

**Table 2:** Association between the main sources of knowledge and the level of knowledge of the Saudi population, a cross-sectional study conducted in Saudi Arabia, June–September 2024 (N=1033)

*Sources of knowledge*	*Level of knowledge*			*p*	*OR*	*95% CI*
	*Good Score ≥16*	*Poor Score ≤15*	*Total*			
	*n (%)*	*n (%)*	*n (%)*			
**Internet**						
Yes	472 (54.1)	400 (45.9)	872 (100)	0.141	1.287	0.92–1.80
No	77 (47.8)	84 (52.2)	181 (100)			
**Social media**						
Yes	416 (55.2)	337 (44.8)	753 (100)	0.027	1.364	1.04–1.80
No	133 (47.5)	147 (52.5)	280 (100)			
**Family and friends**						
Yes	396 (55.8)	314 (44.2)	710 (100)	0.012	1.401	1.08–1.82
No	153 (47.4)	170 (52.6)	323 (100)			
**Printed materials**						
Yes	328 (58.8)	230 (41.2)	558 (100)	<0.001	1.639	1.28–2.10
No	221 (46.5)	254 (53.5)	475 (100)			
**Health staff**						
Yes	436 (57.1)	328 (42.9)	764 (100)	<0.001	1.835	1.39–2.43
No	113 (42.0)	156 (58.0)	269 (100)			
**Radio**						
Yes	322 (56.0)	253 (44.0)	575 (100)	0.039	1.295	1.01–1.86
No	227 (49.6)	231 (50.4)	458 (100)			
**School**						
Yes	381 (59.9)	255 (40.1)	636 (100)	<0.001	2.037	1.58–2.63
No	168 (42.3)	229 (57.7)	397 (100)			
**Other sources**						
Yes	334 (61.5)	209 (38.5)	543 (100)	<0.001	2.044	1.59–2.62
No	215 (43.9)	275 (56.1)	490 (100)			

### Binary logistic regression analysis of variables significantly associated with the level of knowledge

Multivariable binary logistic regression analysis revealed that sex; working in the healthcare field; smoking cigarettes, shisha, or vape; and ‘other sources’ were independently associated with the knowledge level, after adjusting for potential confounders. These associations persisted after adjustments, suggesting that they were not fully explained by the included confounders (Table 3). Men were 0.48 times less likely to achieve a good level of knowledge (95% CI: 0.36–0.65; p<0.001). Healthcare workers had a higher likelihood of achieving good level of knowledge (AOR=2.78; 95% CI: 1.90–4.08; p<0.001). Smokers had lower odds of scoring a good knowledge level than non-smokers (AOR=0.54; 95% CI: 0.38–0.75; p<0.001). The multivariable logistic regression model demonstrated good calibration, as indicated by the non-significant Hosmer-Lemeshow test (p=0.614). The model explained approximately 19.1% of the variance in knowledge levels (Nagelkerke R^2^=0.191; Cox and Snell R^2^=0.143).

**Table 3:** Median split based binary logistic regression analysis of variables significantly associated with the level of knowledge among the Saudi population, a cross-sectional study conducted in Saudi Arabia, June–September 2024 (N=1033)

*Variables*	*Coefficient B*	*p*	*AOR*	*95% CI*
**Age** (years)				
18–25 ®		0.164	1	
26–39	-0.088	0.789	0.92	0.48–1.75
40–50	0.247	0.263	1.28	0.83–1.97
≥51	0.377	0.060	1.46	0.99–2.16
**Sex**	-0.754	<0.001	0.47	0.35–0.64
**Working in the health field**	0.999	<0.001	2.72	1.85–4.00
**Marital status**				
Married ®		0.398	1	
Single	0.320	0.373	1.376	0.68–2.78
Divorced/widowed	0.584	0.183	1.794	0.76–4.24
**Having a fracture**	-0.272	0.070	0.76	0.57–1.02
**Smoking cigarettes, shisha, or vape**	-0.636	<0.001	0.53	0.38–0.74
**Information source**				
Internet	-0.246	0.251	0.78	0.51–1.19
Social media	0.365	0.057	1.44	0.99–2.10
Family and friends	0.309	0.076	1.36	0.97–1.92
Printed materials	0.176	0.267	1.19	0.87–1.63
Health staff	0.190	0.268	1.21	0.86–1.69
Radio	-0.098	0.564	0.91	0.65–1.26
School	0.250	0.132	1.28	0.93–1.78
Other sources	0.641	<0.001	1.90	1.43–2.53
**Constant**	-0.898	0.032	0.41	

AOR: adjusted odds ratio. Hosmer-Lemeshow goodness-of-fit test: p=0.614; Nagelkerke R^2^=0.191; Cox and Snell R²=0.143. ® Reference categories.

### Sensitivity analysis of alternative knowledge-score thresholds

To evaluate the robustness of the findings based on the median cut-off for defining ‘good knowledge’, sensitivity analyses were conducted using alternative thresholds: the upper 33rd percentile (score ≥18) and the upper 25th percentile (score ≥19). When applying the 33rd percentile, multivariable logistic regression showed results consistent with those of the original model. Sex, working in the healthcare field, smoking status, and the use of ‘other sources’ remained significant predictors of knowledge levels with similar ORs and significance levels (Supplementary file Appendix 3). However, when the 25th percentile threshold was applied, only smoking status and ‘other sources’ remained significant, whereas sex and working in the healthcare field were non-significant (Supplementary file Appendix 4). The explanatory power of this model (Nagelkerke R^2^=0.119) was lower than that of the original model (R^2^=0.191). These findings support the median and 33rd percentile thresholds as more stable and meaningful cut-offs for classifying knowledge levels in this population.

## DISCUSSION

The findings of the analysis revealed that the main sources of knowledge on the effects of smoking on fracture healing were the internet and healthcare personnel. In addition, binary logistic regression analysis concluded that men had a 52% lower level of knowledge than women, smokers had a 46% lower level of knowledge than non-smokers, and those working in healthcare had a knowledge level almost three times that of those who were not working in healthcare. Furthermore, of the sources of knowledge, those who chose ‘other sources’ showed a significantly higher level of knowledge.

Previous studies have reported higher rates of smoking in men than in women, similar to that reported in this study^17,18^. Sociocultural norms linking smoking to a masculine image may have been one of the causes of these differences^19^. In addition, cognitive dissonance, a phenomenon in which individuals avoid information that conflicts with their habits, may have affected smokers’ awareness of the impact of smoking on bone healing^20,21^. Meanwhile, female participants demonstrated a higher level of understanding of the effects of smoking on general health, which is consistent with the literature assessing general knowledge of the effects of smoking on health and knowledge of other diseases^22,23^. Despite a Saudi study concluding that men achieved higher levels of perception^24^, limiting participants between 10 and 34 years of age may explain the differences in our outcomes.

Although the initial univariate analysis suggested that a history of fractures and information obtained from healthcare personnel were significantly associated with the level of knowledge, the multivariable binary logistic regression model revealed that both factors could be confounded by other variables.

Although the internet was considered a main source of knowledge by 84% of our cohort, as well as 62% of participants in a local study conducted in the Eastern Province^25^, utilizing it as the primary source of information was not significantly related to the observed level of knowledge. This may be attributed to the lower reliability of health information on the internet. A comprehensive meta-narrative systematic review by Daraz et al.^26^ described online health information as generally suboptimal and unreliable and concluded that the internet is a poor resource in this regard. Similarly, a study by Akerkar et al.^27^ highlighted that the internet significantly influences healthcare decisions, with over 70% of consumers reporting that online information affects their treatment choices. The quality and reliability of this information remain questionable, with most people failing to critically evaluate online health information, often mistakenly perceiving it as trustworthy and of good quality^27^.

A study published in the Saudi Medical Journal stated that 51% of the participants trusted health information from social media platforms. According to the study’s findings, 38% of the participants depended on personal experiences provided on these platforms, although these experiences might not be relevant to others^28^. The implementation of WhatsApp represents the key reason for this particular situation, with governmental reports showing that WhatsApp ranks as the leading social media platform in Saudi Arabia, with 89.9% users^29^. People cannot verify the reliability of health information that appears on WhatsApp. The study demonstrated that Saudi public users shared health messages on WhatsApp groups through which multiple untrustworthy messages could mislead their audience^30^.

Although the results highlight the utilization of multiple sources for obtaining smoking-related health information, such as social media, healthcare personnel, and the internet, a large cohort of participants listed ‘other sources’ as the main source of knowledge of smoking-related health risks. A study conducted in 2023 found that 9% of tobacco smokers visited mobile smoking cessation clinics (SCC) and were counseled on the risks associated with smoking, and 60% of tobacco smokers were aware of SCC^31^. The warning labels displayed on smoking products serve as unspecified sources of knowledge. A European research team found that warning labels directly influence people’s knowledge of smoking risks^32^. Similarly, the Saudi Arabian government adopted plain tobacco packaging while updating cigarette carton warning labels in January 2020^33^. A double-blind clinical trial demonstrated that mobile applications worked effectively to help people quit smoking^34^. Future studies should identify and assess the impact of unspecified sources of awareness on smoking and fracture healing.

### Strengths and limitations

This study assessed awareness of the effects of smoking on fracture healing in a Saudi Arabian population sample. The strengths of this study include a large sample size, use of a validated assessment tool (SFKAT) with excellent reliability, and robust statistical analysis, including binary logistic regression, to adjust for confounders. However, the potential selection bias due to social media recruitment may have overrepresented younger and more educated individuals, which may have overestimated the results. Furthermore, this method may have excluded older populations, rural citizens, and other less digitally connected individuals. Relying on self-reported data may have introduced response bias. In addition, the cross-sectional design only accounts for associations and does not provide causal relationships between the exposure and outcome variables. Although binary logistic regression was implemented to adjust for confounders, residual or unmeasured confounding effects remained unadjusted. The study was conducted in a Saudi Arabian population; hence, the generalizability of the results to other populations may be limited. In addition, the moderate construct validity of some items within the SFKAT was another key limiting factor in this study, as five of the ten items had factor loadings below 0.4, which suggests that these questions may not have a strong alignment with the underlying knowledge construct. This may have lowered the overall precision of the SFKAT in measuring public awareness, potentially diluting the observed associations or misclassifying knowledge levels. To detect subgroup-specific differences in associations, the potential interaction effects (such as between sex and smoking status or sex and working in the healthcare field) need to be analyzed, which were not included in the final regression model. Our primary objective was to identify independent predictors of knowledge. Due to a lack of previous literature and hypotheses, the analysis was performed preliminarily on these factors without testing further subgroups within such factors. Additionally, although the median SFKAT score was used as the main threshold to classify participants into knowledge groups, we conducted a sensitivity analysis using alternative cut-off points (upper 33rd and 25th percentiles) to evaluate the robustness of our classification. The 33rd percentile model yielded results consistent with those of the main model, preserving the significance and direction of the key predictors. However, using the upper 25th percentile as a stricter cut-off altered the statistical significance of some predictors and reduced the model’s explanatory power. These findings support the validity of using the median-based classification while acknowledging that stricter definitions of ‘good knowledge’ may produce less stable models. Future research should address these limitations through more tool refinement and robust population sampling.

## CONCLUSIONS

The knowledge regarding the damaging effects of smoking on fracture healing is suboptimal, especially among men and smokers. Further, sex, healthcare work, and smoking status were determinants of knowledge level. Effective educational programs combined with counseling strategies should be developed to address existing knowledge deficits.

## Supplementary Material



## Data Availability

The data supporting this research are available from the authors on reasonable request.

## References

[1] ElHawary H, Baradaran A, Abi-Rafeh J, Vorstenbosch J, Xu L, Efanov JI. Bone healing and inflammation: principles of fracture and repair. Semin Plast Surg. 2021;35(3):198-203. doi:10.1055/s-0041-1732334 34526868 PMC8432998

[2] Boyette MY, Herrera-Soto JA. Treatment of delayed and nonunited fractures and osteotomies with pulsed electromagnetic field in children and adolescents. Orthopedics. 2012;35(7):e1051-e1055. doi:10.3928/01477447-20120621-20 22784899

[3] Aldhafian M, Alotaibi F, Alzahrani A, et al. Patient-dependent factors for fractures union failure among Riyadh population 2016. J Family Med Prim Care. 2020;9(12):6224-6227. doi:10.4103/jfmpc.jfmpc_1231_20 33681068 PMC7928158

[4] Ekegren CL, Edwards ER, de Steiger R, Gabbe BJ. Incidence, costs and predictors of non-union, delayed union and mal-union following long bone fracture. Int J Environ Res Public Health. 2018;15(12):2845. doi:10.3390/ijerph15122845 30551632 PMC6313538

[5] Hak DJ, Fitzpatrick D, Bishop JA, et al. Delayed union and nonunions: epidemiology, clinical issues, and financial aspects. Injury. 2014;45(Suppl 2):S3-S7. doi:10.1016/j.injury.2014.04.002 24857025

[6] Silverstein P. Smoking and wound healing. Am J Med. 1992;93(1A):22S-24S. doi:10.1016/0002-9343(92)90623-j 1323208

[7] Nunna RS, Ostrov PB, Ansari D, et al. The Risk of nonunion in smokers revisited: a systematic review and meta-analysis. Global Spine J. 2022;12(3):526-539. doi:10.1177/21925682211046899 34583570 PMC9121161

[8] Agoons DD, Agoons BB, Emmanuel KE, Matawalle FA, Cunningham JM. Association between electronic cigarette use and fragility fractures among US adults. Am J Med Open. 2021;1-6:100002. doi:10.1016/j.ajmo.2021.100002 39036626 PMC11256257

[9] Ministry of Health, Saudi Arabia. GATS KSA, 2019: Global Adult Tobacco Survey. Ministry of Health, Saudi Arabia. 2019. Accessed May 28, 2024. https://www.moh.gov.sa/en/Ministry/Statistics/Population-Health-Indicators/Documents/KSA_GATS_2019_FactSheet.pdf

[10] Alotaibi SA, Alsuliman MA, Durgampudi PK. Smoking tobacco prevalence among college students in the Kingdom of Saudi Arabia: systematic review and meta-analysis. Tob Induc Dis. 2019;17:35. doi:10.18332/tid/105843 31516478 PMC6662783

[11] Tobacco. World Health Organization. Accessed August 7, 2025. https://www.who.int/news-room/fact-sheets/detail/tobacco

[12] Chang CJ, Jou IM, Wu TT, Su FC, Tai TW. Cigarette smoke inhalation impairs angiogenesis in early bone healing processes and delays fracture union. Bone Joint Res. 2020;9(3):99-107. doi:10.1302/2046-3758.93.BJR-2019-0089.R1 32435462 PMC7229299

[13] Patel RA, Wilson RF, Patel PA, Palmer RM. The effect of smoking on bone healing: a systematic review. Bone Joint Res. 2013;2(6):102-111. doi:10.1302/2046-3758.26.2000142 23836474 PMC3686151

[14] Ward KD, Klesges RC. A meta-analysis of the effects of cigarette smoking on bone mineral density. Calcif Tissue Int. 2001;68(5):259-270. doi:10.1007/BF02390832 11683532 PMC5352985

[15] Nguyen VH. Smoking status on bone health and osteoporosis prevalence. Osong Public Health Res Perspect. 2018;9(4):213-214. doi:10.24171/j.phrp.2018.9.4.11 30159228 PMC6110322

[16] Alshammari MB, Haridi HK. Prevalence and determinants of exclusive breastfeeding practice among mothers of children aged 6-24 months in Hail, Saudi Arabia. Scientifica. 2021;2021:2761213. doi:10.1155/2021/2761213 33854807 PMC8019643

[17] Institute for Health Metrics and Evaluation. Smoking in the Kingdom of Saudi Arabia: Findings from the Saudi health interview survey. Accessed June 13, 2025. https://www.healthdata.org/sites/default/files/files/Projects/KSA/Smoking-KSA-Findings-from-the-Saudi-Health-Interview-Survey.pdf

[18] Algabbani AM, Almubark RA, Althumiri NA, Alqahtani AS, BinDhim NF. The prevalence of cigarette smoking in Saudi Arabia in 2018. Food and Drug Regulatory Science Journal. 2018;1(1):1.

[19] Abdalla AM, Al-Kaabba AF, Saeed AA, Abdulrahman BM, Raat H. Gender differences in smoking behavior among adolescents in Saudi Arabia. Saudi Med J. 2007;28(7):1102-1108. 17603720

[20] Fotuhi O, Fong GT, Zanna MP, Borland R, Yong HH, Cummings KM. Patterns of cognitive dissonance-reducing beliefs among smokers: a longitudinal analysis from the International Tobacco Control (ITC) four country survey. Tob Control. 2013;22(1):52-58. doi:10.1136/tobaccocontrol-2011-050139 22218426 PMC4009366

[21] Orcullo DJC, Teo HS. Understanding cognitive dissonance in smoking behavior: a qualitative study. Int J Soc Sci Humanit. 2016;6(6):481-485. doi:10.7763/IJSSH.2016.V6.695

[22] Ahmad W, Ahmad A, Ali MD, et al. Cross sectional online survey to determine the prevalence, knowledge, attitude and practice of smoking tobacco among students of medical science college in Dammam, Saudi Arabia. J Pharm Bioallied Sci. 2021;13(3):305-311. doi:10.4103/jpbs.jpbs_28_21 35017886 PMC8698083

[23] Aljefree NM, Almoraie NM, Althaiban MA, Hanbazaza MA, Wazzan HA, Shatwan IM. Gender differences in knowledge, attitudes, and practices with respect to type 1 diabetes among Saudi public-school teachers. BMC Public Health. 2023;23(1):118. doi:10.1186/s12889-023-15043-w 36650460 PMC9847176

[24] Alarfaj MO, Alshammari MM, Albenayyan HA, Alonazi AA, Alkhateeb AA, Al Taisan A. Awareness of blindness related to smoking among young age population: a cross-sectional study in Saudi Arabia. Cureus. 2022;14(10):e30501. doi:10.7759/cureus.30501 36415373 PMC9674951

[25] AlJumaan M, Aldajani A, Al Jamaan Y, Alawami A, Alarfaj M, Alkhadra F. Effect of health information websites on healthcare facility visits in the eastern province of Saudi Arabia. Integrative Journal of Medical Sciences. 2020;7:166. doi:10.15342/ijms.7.166

[26] Daraz L, Morrow AS, Ponce OJ, et al. Can patients trust online health information? A meta-narrative systematic review addressing the quality of health information on the internet. J Gen Intern Med. 2019;34(9):1884-1891. doi:10.1007/s11606-019-05109-0 31228051 PMC6712138

[27] Akerkar SM, Bichile LS. Health information on the internet: patient empowerment or patient deceit? Indian J Med Sci. 2004;58(8):321-326. 15345885

[28] Sumayyia MD, Al-Madaney MM, Almousawi FH. Health information on social media. Perceptions, attitudes, and practices of patients and their companions. Saudi Med J. 2019;40(12):1294-1298. doi:10.15537/smj.2019.12.24682 31828284 PMC6969626

[29] Saudi Communications, Space & Technology Commission. Saudi internet. Saudi Communications, Space & Technology Commission website. 2021. Accessed December 28, 2024. https://www.cst.gov.sa/ar/indicators/PublishingImages/Pages/saudi_internet/internt-saudi-2021.pdf

[30] Alfaris E, Alhazzani Y, Alkhenizan A, et al. Assessing the validity of health messages used by the Saudi public in Whatsapp. Patient Prefer Adherence. 2023;17:67-73. doi:10.2147/PPA.S397661 36632071 PMC9829413

[31] Monshi SS, Alanazi AMM, Alzahrani AM, et al. Awareness and utilization of smoking cessation clinics in Saudi Arabia, findings from the 2019 Global Adult Tobacco Survey. Subst Abuse Treat Prev Policy. 2023;18(1):33. doi:10.1186/s13011-023-00543-0 37322497 PMC10268372

[32] Trofor AC, Papadakis S, Lotrean LM, et al. Knowledge of the health risks of smoking and impact of cigarette warning labels among tobacco users in six European countries: findings from the EUREST-PLUS ITC Europe Surveys. Tob Induc Dis. 2019;16:A10. doi:10.18332/tid/99542 31516464 PMC6661855

[33] Campaign for Tobacco-Free Kids. Standardized or plain tobacco packaging: international developments. Campaign for Tobacco-Free Kids. Updated December 2023. Accessed November 17, 2024. https://assets.tobaccofreekids.org/global/pdfs/en/standardized_packaging_developments_en.pdf

[34] Bricker JB, Watson NL, Mull KE, Sullivan BM, Heffner JL. Efficacy of smartphone applications for smoking cessation: a randomized clinical trial. JAMA Intern Med. 2020;180(11):1472-1480. doi:10.1001/jamainternmed.2020.4055 32955554 PMC7506605

