# Supplementary material for: Awareness of the Saudi population regarding the effects of smoking on fracture healing
Source: Tob Induc Dis. 2025 Aug 21;23:10.18332/tid/207130. doi: 10.18332/tid/207130 (PMC12371527; doi:10.18332/tid/207130)
Supplement: Supplementary file 1 [file TID-23-118-s1.pdf]

Supplementary materials for the article titled: **Awareness of the Saudi population regarding the effects of smoking on fracture healing**

Tables:

Appendix 1. Smoking and Fractures Knowledge Assessment Tool (SFKAT)[a] in a cross-sectional study conducted in Saudi Arabia (N=1033), June–September 2024

	**Question**	**True**	**False**	**Not sure**
14	Smoking delays fracture healing time.			
15	Smoking causes failure of fracture to heal properly.			
16	Smoking increases the risk of deep infections at the fracture site.			
17	Smoking reduces bone mineral density.			
18	Smoking impairs the cells responsible for bone formation.			
19	Smoking increases the risk of complications after surgery.			
20	Smoking impairs the bone regeneration process.			
21	Most of the effects of smoking on bones and fractures are caused by nicotine.			
22	Nicotine-free smoking products have a lower risk of fracture-related complications.			
23	Prolonged cessation of smoking before surgery can significantly reduce the risk of complications.			

a. The total score is 20. Two marks for choosing "True", one mark for "Not sure", and zero marks for choosing "False" as an answer to each question.

Appendix 2. Demographic characteristics of participants in a cross-sectional study conducted in Saudi Arabia (N=1033), June–September 2024

**Sociodemographics**	**Variables**	***n* (%)**
Age groups	18-25<br>26-39<br>40-50	262 (25.4)<br>263 (25.5)<br>277 (26.8)

**Sociodemographics**	**Variables**	***n* (%)**
	51 or older	231 (22.3)
Sex	Male<br>Female	562 (54.4)<br>471 (45.6)
Education	High school or lower<br>Diploma/Bachelor's<br>Higher education	116 (11.2)<br>697 (67.5)<br>220 (21.3)
Working in the health field	Yes<br>No	209 (20.2)<br>824 (79.8)
Marital status	Married<br>Single<br>Divorced/Widowed	645 (62.4)<br>350 (33.9)<br>38 (3.7)
Saudi Arabia Regions	Central<br>Northern<br>Southern<br>Eastern<br>Western	649 (62.8)<br>24 (2.3)<br>77 (7.5)<br>111 (10.7)<br>172 (16.7)
Living area	City<br>Governorate<br>Village	920 (89.1)<br>76 (7.4)<br>37 (3.6)
Employment	Employed<br>Unemployed<br>Freelancer<br>Retired<br>Student<br>Other	538 (52.1)<br>105 (10.2)<br>23 (2.2)<br>165 (16)<br>178 (17.2)<br>24 (2.3)
Income	Less than 5000 SAR<br>5000-9999 SAR	61 (5.9)<br>136 (13.2)

Sociodemographics	Variables	*n* (%)
	10000-14999 SAR<br>15000-19999 SAR<br>20000-40000 SAR<br>More than 40000 SAR	213 (20.6)<br>230 (22.3)<br>248 (24)<br>145 (14)
Did you have a fracture	Yes<br>No	301 (29.1)<br>732 (70.9)
Smoke cigarettes, or shisha, or vape	Yes<br>No	249 (24.1)<br>784 (75.9)

Appendix 3.The 33th upper percentile based Binary logistic regression analysis of variables significantly associated with the level of knowledge among the Saudi Population in a cross-sectional study conducted in Saudi Arabia (N=1033), June–September 2024[a]

Associated variables	Coefficient B	p-value	Adjusted odds ratio aOR	95% CI[b]
18-25	Reference	0.080	Reference	Reference
26-39	0.183	0.578	1.20	0.63 - 2.28
40-50	0.444	0.049	1.56	1.00 - 2.43
51 or older	0.470	0.022	1.60	1.07 - 2.39
Sex	-0.340	0.025	0.71	0.53 - 0.96
Working in the health field	0.476	0.011	1.61	1.12 - 2.32
Married	Reference	0.835	Reference	Reference
Single	0.220	0.550	1.25	0.61 - 2.56
Divorced/Widowed	0.227	0.608	1.26	0.53 - 2.99

Having a fracture	0.008	0.957	1.01	0.75 - 1.36
Smoking cigarettes, shisha, or vape	-0.878	<0.001	0.42	0.29 - 0.60
Internet	-0.135	0.541	0.87	0.57 - 1.35
Social media	0.029	0.880	1.03	0.70 - 1.50
Family and Friends	0.391	0.029	1.48	1.04 - 2.10
Printed materials	0.246	0.124	1.28	0.94 - 1.75
Health staff	-0.039	0.825	0.96	0.68 - 1.36
Radio	-0.061	0.723	0.94	0.67 - 1.32
School	0.431	0.012	1.54	1.10 - 2.15
Other sources	0.583	<0.001	1.79	1.34 - 2.39
Constant	-1.597	<0.001	0.20	

a. Hosmer–Lemeshow goodness-of-fit test: $p = 0.264$; Nagelkerke $R^2 = 0.141$; Cox & Snell $R^2 = 0.104$.
b. CI: Confidence interval.

Appendix 4. The 25th upper percentile based Binary logistic regression analysis of variables significantly associated with the level of knowledge among the Saudi Population in a cross-sectional study conducted in Saudi Arabia (N=1033), June–September 2024[a]

Associated variables	Coefficient B	p-value	Adjusted odds ratio aOR	95% CI[b]
18-25	Reference	0.022	Reference	Reference
26-39	0.533	0.133	1.70	0.85 - 3.42
40-50	0.701	0.004	2.02	1.25 - 3.26

51 or older	0.579	0.01	1.78	1.15 - 2.77
Sex	-0.232	0.153	0.79	0.58 - 1.09
Working in the health field	0.085	0.674	1.09	0.73 - 1.61
Married	Reference	0.302	Reference	Reference
Single	0.573	0.178	1.77	0.77 - 4.08
Divorced/Widowed	0.339	0.498	1.40	0.53 - 3.75
Having a fracture	0.043	0.794	1.04	0.76 - 1.48
Smoking cigarettes, shisha, or vape	-0.833	<0.001	0.44	0.29 - 0.66
Internet	-0.284	0.238	0.73	0.47 - 1.21
Social media	0.191	0.368	1.21	0.80 - 1.84
Family and Friends	0.268	0.173	1.31	0.89 - 1.92
Printed materials	0.189	0.275	1.21	0.86 - 1.70
Health staff	0.096	0.626	1.10	0.75 - 1.62
Radio	0.017	0.927	1.01	0.71 - 1.46
School	0.484	0.010	1.62	1.12 - 2.35
Other sources	0.540	0.001	1.72	1.25 - 2.35
Constant	-2.587	<0.001	0.08	

a. Hosmer–Lemeshow goodness-of-fit test: $p = 0.801$; Nagelkerke $R^2 = 0.119$; Cox & Snell $R^2 = 0.082$.

b. CI: Confidence interval.

Figure 1. Distribution of total SFKAT scores among 1033 participants in a cross-sectional study conducted in Saudi Arabia (N=1033), June–September 2024.

Figure 1. Distribution of total SFKAT scores among 1033 participants in a cross-sectional study conducted in Saudi Arabia (N=1033), June–September 2024.

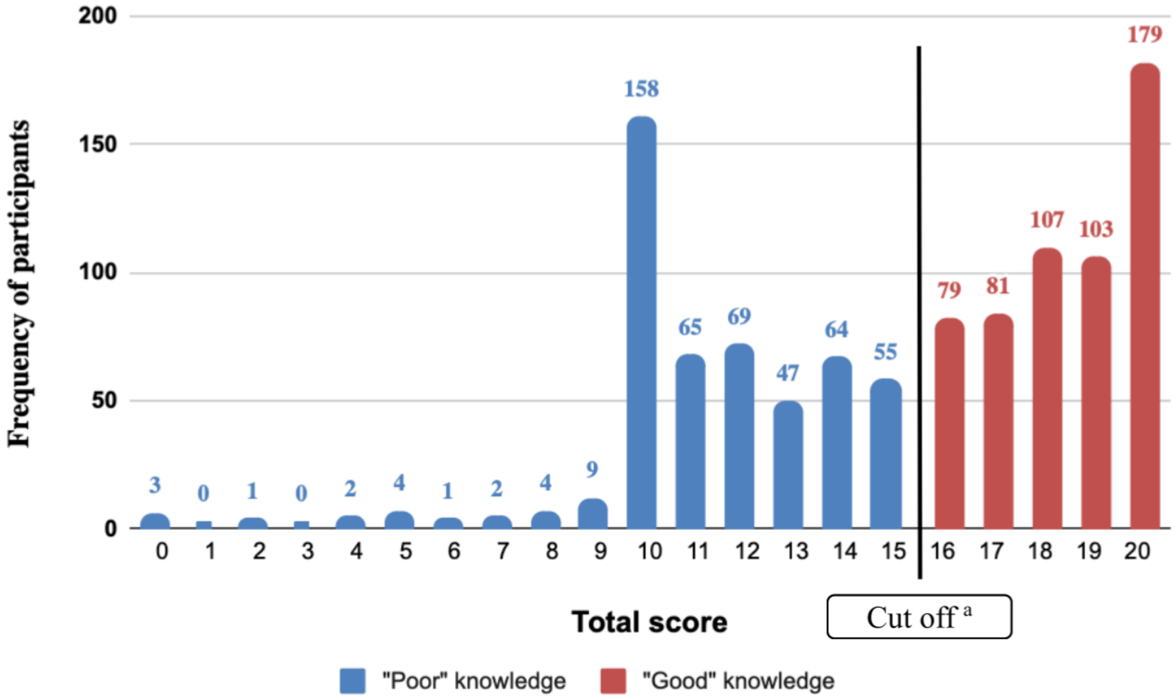

a. This line represents the cutoff point for knowledge level; scores above it indicate good knowledge, while scores below it reflect poor knowledge.

Figure 2. Frequency of choosing the main sources of knowledge in a cross-sectional study conducted in Saudi Arabia (N=1033), June–September 2024

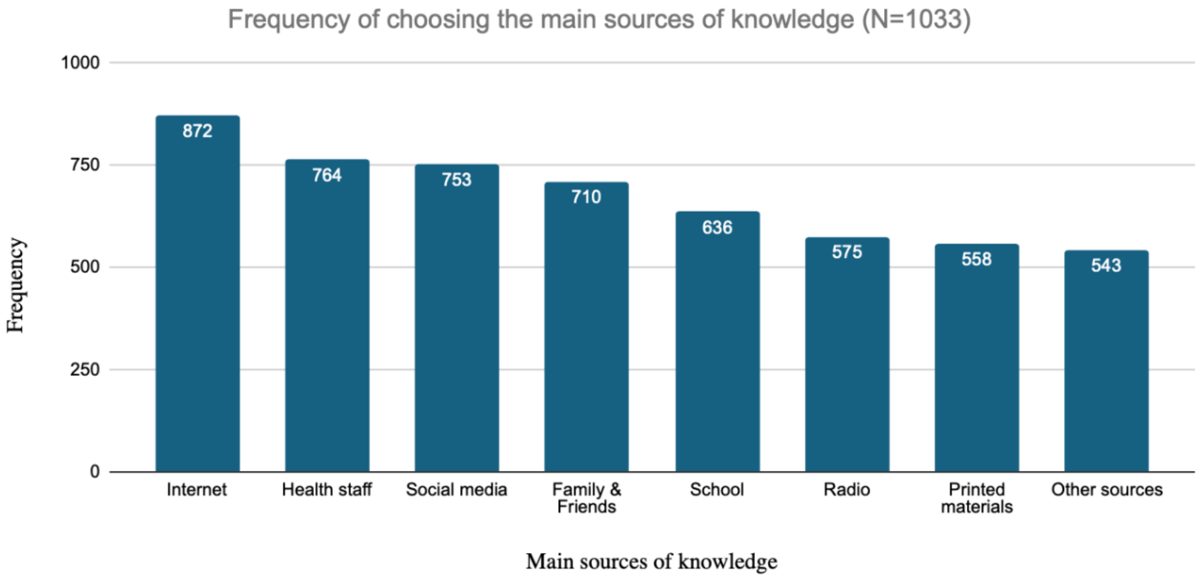

Table 1. Association between the dichotomous sociodemographic characteristics and the level of knowledge of the Saudi Population in a cross-sectional study conducted in Saudi Arabia (N=1033), June–September 2024.

**Sociodemographic characteristics**		**Level of knowledge**			**p-value**	**OR[a]**	**95% CI[b]**
		**Good Score ≥ 16**	**Poor Score ≤ 15**	**Total**			
		n (%)	***n (%)***	***n (%)***			
Sex	Male	245 (43.6)	317 (56.4)	562 (100)	<0.001	0.43	0.33 - 0.55
	Female	304 (64.5)	167 (35.5)	471 (100)			
Working in the health field	Yes	144 (68.9)	65 (31.1)	209 (100)	<0.001	2.29	1.66 - 3.17
	No	405 (49.2)	419 (50.8)	824 (100)			
Having a fracture	Yes	142 (47.2)	159 (52.8)	301 (100)			

	No	407 (55.6)	325 (44.4)	732 (100)	0.014	0.71	0.55 - 0.93
Smoking cigarettes, shisha, or vape	Yes	88 (35.3)	161 (64.7)	249 (100)	<0.001	0.38	0.29 - 0.52
	No	461 (58.8)	323 (41.2)	784 (100)			

a. OR: Odds Ratio.

b. CI: Confidence interval.

Table 2. Association between the main sources of knowledge and the level of knowledge of the Saudi Population in a cross-sectional study conducted in Saudi Arabia (N=1033), June–September 2024.

Sources of knowledge		Level of knowledge			p-value	Odds Ratio	95% CI[a]
		Good Score ≥ 16	Poor Score ≤ 15	Total			
		n (%)	*n (%)*	*n (%)*			
Internet	Yes	472 (54.1)	400 (45.9)	872 (100)			
	No	77 (47.8)	84 (52.2)	181 (100)	0.141	1.287	(0.92 - 1.80)
Social media	Yes	416 (55.2)	337 (44.8)	753 (100)			
	No	133 (47.5)	147 (52.5)	280 (100)	0.027	1.364	(1.04 - 1.80)
Family and Friends	Yes	396 (55.8)	314 (44.2)	710 (100)			
	No	153 (47.4)	170 (52.6)	323 (100)	0.012	1.401	(1.08 - 1.82)
Printed materials	Yes	328 (58.8)	230 (41.2)	558 (100)			
	No	221 (46.5)	254 (53.5)	475 (100)	<0.001	1.639	(1.28 - 2.10)
Health staff	Yes	436 (57.1)	328 (42.9)	764 (100)			

	No	113 (42.0)	156 (58.0)	269 (100)	<0.001	1.835	(1.39 - 2.43)
Radio	Yes<br>No	322 (56.0)<br>227 (49.6)	253 (44.0)<br>231 (50.4)	575 (100)<br>458 (100)	0.039	1.295	(1.01 - 1.86)
School	Yes<br>No	381 (59.9)<br>168 (42.3)	255 (40.1)<br>229 (57.7)	636 (100)<br>397 (100)	<0.001	2.037	(1.58 - 2.63)
Other sources	Yes<br>No	334 (61.5)<br>215 (43.9)	209 (38.5)<br>275 (56.1)	543 (100)<br>490 (100)	<0.001	2.044	(1.59 - 2.62)

a. CI: Confidence interval.

Table 3: Median split based Binary logistic regression analysis of variables significantly associated with the level of knowledge among the Saudi Population in a cross-sectional study conducted in Saudi Arabia (N=1033), June–September 2024[a]

Associated variables	Coefficient B	p-value	Adjusted odds ratio aOR	95% CI[b]
18-25	Reference	0.164	Reference	Reference
26-39	-0.088	0.789	0.92	0.48 - 1.75
40-50	0.247	0.263	1.28	0.83 - 1.97
51 or older	0.377	0.060	1.46	0.99 - 2.16
Sex	-0.754	<0.001	0.47	0.35 - 0.64
Working in the health field	0.999	<0.001	2.72	1.85 - 4.00
Married	Reference	0.398	Reference	Reference
Single	0.320	0.373	1.376	0.68 - 2.78
Divorced/Widowed	0.584	0.183	1.794	0.76 - 4.24

Having a fracture	-0.272	0.070	0.76	0.57 - 1.02
Smoking cigarettes, shisha, or vape	-0.636	<0.001	0.53	0.38 - 0.74
Internet	-0.246	0.251	0.78	0.51 - 1.19
Social media	0.365	0.057	1.44	0.99 - 2.10
Family and Friends	0.309	0.076	1.36	0.97 - 1.92
Printed materials	0.176	0.267	1.19	0.87 - 1.63
Health staff	0.190	0.268	1.21	0.86 - 1.69
Radio	-0.098	0.564	0.91	0.65 - 1.26
School	0.250	0.132	1.28	0.93 - 1.78
Other sources	0.641	<0.001	1.90	1.43 - 2.53
Constant	-0.898	0.032	0.41	

a. Hosmer–Lemeshow goodness-of-fit test: $p = 0.614$; Nagelkerke $R^2 = 0.191$; Cox & Snell $R^2 = 0.143$.
b. CI: Confidence interval.

The questionnaire:

First: Sociodemographic Characteristics

1. Age:
 Write your age (number): _____

2. Sex:
- Male
- Female

3. Educational Level:

- High school or lower
- Diploma/Bachelor's
- Higher Education

4. Field of Work/Study

5. Marital Status:
   - Married
   - Single
   - Divorced/Widowed

6. Where do you live in Saudi Arabia?
   - Central Region
   - Northern Region
   - Southern Region
   - Eastern Region
   - Western Region

7. Where do you live?
   - City
   - Governorate
   - Village

8. Occupational Status:
   - Employed
   - Unemployed
   - Freelancer
   - Retired
   - Student
   - Other

9. Total Monthly Household Income:

- Less than 5000 SAR
- Between 5000 – 9999 SAR
- Between 10000 – 14999 SAR
- Between 15000 – 19999 SAR
- Between 20000 – 40000 SAR
- More than 40000 SAR

10. Nationality:

- Saudi
- Non-Saudi

11. Type of Latest Fracture happened to you:

- Lower limb fracture (legs)
- Upper limb fracture (arms)
- Isolated pelvic fracture
- Multiple fractures at once
- Never had a fracture
- Other

12. If you smoke cigarettes, for how many years have you been smoking?

"If you do not smoke cigarettes, write 0"

Write number of years (example, 3): _____

13. If you smoke hookah (shisha), for how many years have you been smoking?

"If you do not smoke hookah (shisha), write 0"

Write number of years (example, 3): _____

14. If you use vape (electronic shisha), for how many years have you been using it?

"If you do not use vape (electronic shisha), write 0"

Write number of years (example, 3): _____

Second: Knowledge Assessment on the Effects of Smoking on Fracture Healing

	Question	True	False	Not sure
15	Smoking delays fracture healing time.			
16	Smoking causes failure of fracture to heal properly.			
17	Smoking increases the risk of deep infections at the fracture site.			
18	Smoking reduces bone mineral density.			
19	Smoking impairs the cells responsible for bone formation.			
20	Smoking increases the risk of complications after surgery.			
21	Smoking impairs the bone regeneration process.			
22	Most of the effects of smoking on bones and fractures are caused by nicotine.			
23	Nicotine-free smoking products have a lower risk of fracture-related complications.			
24	Prolonged cessation of smoking before surgery can significantly reduce the risk of complications.			

Third: Main Sources of Knowledge on the Effects of Smoking on Fracture Healing

Which of the following sources do you use to gain knowledge about the effects of smoking on fracture healing?

25. Internet Navigation:

- Yes
- No

26. Social media:

- Yes
- No

27. Family/friends:

- Yes
- No

28. Printed materials (pamphlets, banners, Other materials.):
- Yes
- No

29. Healthcare staff:
- Yes
- No

30. Radio/television:
- Yes
- No

31. School/work:
- Yes
- No

32. Other sources:
- Yes
- No

© 2025 Alrabai H.M. et al.
